# No Alteration of Optical Coherence Tomography and Multifocal Visual Evoked Potentials in Eyes With Symptomatic Carotid Artery Disease

**DOI:** 10.3389/fneur.2019.00741

**Published:** 2019-07-10

**Authors:** John-Ih Lee, Lena Gemerzki, Laura Boerker, Rainer Guthoff, Orhan Aktas, Michael Gliem, Sebastian Jander, Hans-Peter Hartung, Philipp Albrecht

**Affiliations:** ^1^Department of Neurology, Medical Faculty, Heinrich-Heine-University, Duesseldorf, Germany; ^2^Department of Ophthalmology, Medical Faculty, Heinrich-Heine-University, Duesseldorf, Germany

**Keywords:** retinal layer, optical coherence tomography, multifocal visual evoked potential, symptomatic carotid artery disease, ischemic stroke

## Abstract

**Background:** Symptomatic carotid artery disease (CAD) may cause modified blood supply to the retina possibly leading to retinal structure changes. Results of previous studies in asymptomatic CAD were heterogeneous in retinal layer changes measured by OCT. The objectives of this prospective, non-interventional study were to investigate if structural retinal changes occur in symptomatic CAD patients with macroangiopathic ischemic stroke or transient ischemic attack (TIA).

**Methods:** We used spectral-domain optical coherence tomography (SD-OCT) to cross-sectionally and longitudinally analyze the retinal morphology of CAD patients with macroangiopathic ischemic stroke or TIA not permanently affecting the visual pathway. We employed semi-automated segmentation of macular volume scans to assess the macular retinal layers' thickness and peripapillary ring scans to determine the peripapillary retinal nerve fiber layer thickness using the contralateral eye and eyes of microangiopathic ischemic stroke patients with matched age, gender, and vascular risk factors as control. Visual function and visual field deficits were assessed by multifocal visual evoked potentials (mfVEP).

**Results:** Neither the thickness of retinal layers measured by SD-OCT in 17 patients nor the mfVEP latency or amplitude in 10 patients differed between the symptomatic stenotic, the contralateral internal carotid artery (ICA) side and the control group of 17 microangiopathic stroke patients at baseline. Furthermore, longitudinal investigations of 10 patients revealed no significant changes of any retinal layer 4 months after ischemic stroke or TIA.

**Conclusion:** In conclusion, our study revealed no evidence for an impact of symptomatic carotid artery disease on retinal structure or functional impairment of the visual pathway.

## Introduction

Ischemic stroke is among the most common reasons for years of life lost (YLL) (1). Macroangiopathic extracranial internal carotid artery stenosis is a major risk factor for ischemic stroke. As there are effective treatment options for symptomatic moderate to high grade internal carotid artery stenoses including carotid endarterectomy, stent assisted carotid angioplasty and medical therapy, detection of ICA stenosis is important for stroke prevention. Furthermore, symptomatic ICA stenosis may be associated with additional, more subtle pathologies than stroke which could be of prognostic value and clinical relevance. The ICA supplies the retina with blood via the ophthalmic artery. In high grade ICA stenosis flow reversal of the supraorbital and supratrochlear artery can occur, and in this case the ophthalmic artery can be supplied by collateral pathways from the external carotid artery. We hypothesized that modified blood flow of the ICA might cause structural and/or functional abnormalities of the retina or visual pathway below the threshold of permanent clinical symptoms. Structural abnormalities of the retinal layers can be non-invasively detected by spectral domain optical coherence tomography (SD-OCT). SD-OCT allows a reliable measurement of the retinal layers and can detect retinal axonal and neuronal loss even in the absence of overt visual symptoms in other diseases (2–4). Functional impairment of the visual pathway can be assessed by visual evoked potentials (VEPs) (5–7), and multifocal VEPs (mfVEPs) have demonstrated a higher sensitivity for pathology compared to fullfield VEPs (5–7). The aim of our study was to cross-sectionally and longitudinally analyze subclinical differences in retinal layers or visual function in eyes ipsilateral to symptomatic internal carotid artery stenoses ≥ 50% without permanent clinical deficit in the visual pathway.

## Patients and Methods

### Ethics

The local ethics committee of Heinrich Heine University Duesseldorf approved this prospective observational study (Study-number 4436R). Written informed consent was obtained from all participants in accordance with the Declaration of Helsinki.

### Patients

Patients were prospectively recruited from the stroke unit and followed up in the outpatient clinic at the Department of Neurology, Heinrich Heine University Duesseldorf, Germany from 2013 to 2016. Inclusion criteria were ischemic stroke or transient ischemic attack (TIA) in the internal carotid artery system without permanently affecting the visual pathway, and ipsilateral ≥ 50% internal carotid artery stenosis detected by multiparametric “Deutsche Gesellschaft für Ultraschall in der Medizin” (DEGUM) ultrasound criteria according to the NASCET definition (8). All patients underwent a neuro-ophthalmologic examination including slit lamp examination, tonometry and ophthalmoscopic fundus imaging. Exclusion criteria were relevant ophthalmologic and systemic diseases with potential influence on retinal morphology as defined by the OSCAR-IB criteria (9) and older cerebral lesions within the visual pathway. Corrected visual acuity was assessed using ETDRS charts.

**Figure 1:**
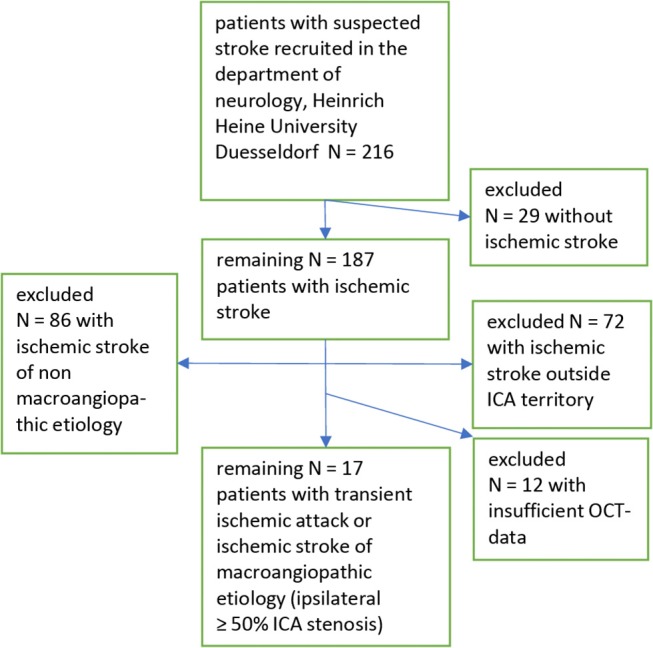
Flow chart of the inclusion/exclusion process.

Out of 216 patients recruited from our stroke unit at the Heinrich Heine University Düsseldorf 17 patients with symptomatic internal carotid artery stenosis ≥50% remained in our analysis. For details see the recruitment flow chart (Figure 1).

The sCAD group was compared to a control group of 17 patients with microangiopathic ischemic strokes according to the Trial of Org 10172 in Acute Stroke Treatment (TOAST) classification (10). None of the patients of our microangiopathic ischemic stroke control group had an affection of the visual pathway, neither clinically nor detected by imaging in computed tomography (CT) or magnetic resonance imaging (MRI).

### Ultrasound

The extra- and intracranial brain supplying arteries of all patients were examined by ultrasound using a Toshiba ultrasound system (Aplio XG, Xario) and DWL Multi Dop Pro. Extracranial flow velocities were measured with the linear probe (7.5 MHz), intracranial vessels with a sector probe (2.5 MHz). The flow curves of the supratrochlear and supraorbital arteries were recorded with pen probe doppler (8 MHz), the flow direction was detected by sequential compression tests of the superficial temporal and facial arteries bilaterally as described before (11). The ICA stenosis was classified according to the multiparametric DEGUM ultrasound criteria (8). Intima media thickness (IMT) was obtained at the far wall side of both common carotid arteries (CCA) 2 cm below the distal end of CCA (12, 13).

### OCT Methodology

Optical coherence tomography methodology and results are reported in line with the APOSTEL reporting recommendations (14). We obtained volumetric retinal scans consisting of 61 vertical scans centered on the fovea (30° ×25°, high speed scanning mode) as well as 12° peripapillary ring scans centered on the disc (high-resolution scanning mode) using a SPECTRALIS OCT device (Heidelberg Engineering, Germany) with image alignment eye-tracking software (TruTrack and Nsite analytics, Heidelberg Engineering) of both eyes. All scans were performed with support of the eye-tracking system by trained technicians. The macular volume scans were averaged from 14 images, while the peripapillary ring scans were averaged from 100 scans (Automatic Real Time, ART). The image quality of all scans was above 20 dB. Segmentation of retinal layers was performed semi-automatically with manual correction of errors using the Heidelberg Eye Explorer software (version HEYEX 1.8.6.0, Viewing Module 5.8.3.0). Due to the low contrast between the ganglion cell layer (GCL) and the inner plexiform layer (IPL), both layers were measured together as the ganglion cell/inner plexiform layer complex (GCIP). All scans were checked for correct segmentation and segmentation errors were corrected manually by a blinded rater (LG). Scans not meeting the OSCAR-IB quality control criteria (9) were discarded. Therefore, not all scans of all patients were available for analysis. We indicate the layer volumes of the retinal layers as well as the total retinal volume (TRV), measured using the mean volume of all sectors of the standard 1, 3, 6 mm ETDRS (early treatment of diabetic retinopathy study) grid in macular volume scans.

### MfVEP Methodology

MfVEPs were recorded to assess the function of the visual pathway using a Visionsearch mfVEP device according to the manufacturer's instructions as previously described (15). In short, monocular stimulation was performed using the Visionsearch device applying simultaneous multi-focal stimulation of 56 segments of the visual field (24 degrees of eccentricity) via a 68 s pseudorandom sequence and recording a 2-channel visual response using a custom designed occipital cross electrode holder which predetermines the four occipital electrode positions (16). The checkerboard pattern used for stimulation was scaled based on cortical magnification. To determine sufficient amplitudes, the software automatically calculated the signal to noise ratio at each segment of the stimulated visual field (17). The amplitude of multiple segments added after cross-correlation was performed and individual traces were defined, avoiding cancellation of the dipoles, which often occurs in full-field VEP (15, 16). Mean values of both amplitude and latency, which were used in the final analysis, were calculated by averaging the amplitude and latency of the individual sectors as previously described (15, 16).

### Statistical Evaluation

Statistical analyses were performed using SPSS Statistics 20 (IBM). Follow up investigations were performed between 2 and 6 months, and the values were normalized to 4 months for statistical analysis. Due to the small group sizes the non-parametric Wilcoxon matched pairs test was performed to test for differences of OCT parameters and mfVEP parameters at baseline and the change rates at the 4-month follow up between the eye located on the side of the symptomatic stenotic ICA, which supplied the vascular territory of the ischemic stroke or TIA and the contralateral eye. The Mann Whitney U test was performed to compare these parameters of the symptomatic stenotic ICA side, the contralateral asymptomatic side, and the mean of the right and the left eyes of the control group with microangiopathic ischemic stroke. Nominally scaled baseline parameters were compared by Chi Square test. As several retinal layers, mfVEP amplitudes and latencies were analyzed in this exploratory analysis, Bonferroni correction for multiple testing was performed. Spearman correlation analysis was performed to investigate the association between IMT and retinal layers. Subjects with missing data were excluded from the respective analysis, *p*-values < 0.05 were considered significant.

## Results

### Patients

Seventeen patients with sCAD were included according to our inclusion and exclusion criteria. Furthermore, for comparison 17 control patients with microangiopathic ischemic stroke according to (TOAST) classification (10) with <50% bilateral ICA stenosis were analyzed. The control group with microangiopathic ischemic stroke showed statistically no difference to the sCAD group in age, gender, time from ischemic event to baseline OCT and mfVEP assessment, and vascular risk factor profile, except for hyperlipidemia, which was more frequent in the sCAD group. Baseline parameters, vascular risk factors and time period of initial investigations including OCT und mfVEP after the ischemic stroke or TIA event of both groups are presented in Table 1. In the sCAD group 8 (47%) patients had an ischemic stroke in the territory of the middle cerebral artery, 5 (29%) patients had a TIA in the territory of the middle cerebral artery, and 4 (24%) patients had an amaurosis fugax attack.

**Table 1:** Baseline parameters of the 17 patients with symptomatic ≥ 50% ICA stenosis and 17 control group patients with microangiopathic ischemic stroke.

	**sCAD group, *N* = 17**	**Control group with microangiopathic ischemic stroke, *N* = 17**	
Median age in years (interquartile range)	64 (60–74)	64 (53.5–72)	Mann Whitney *U*-test n.s.
Male gender	12 (71%)	12 (71%)	Chi Square test n.s.
Arterial hypertension	12 (71%)	14 (84%)	Chi Square test n.s.
Diabetes mellitus	3 (18%)	6 (35%)	Chi Square test n.s.
Vascular disease	4 (24%)	4 (24%)	Chi Square test n.s
Smoking	11 (65%)	11 (65%)	Chi Square test n.s.
Coronary heart disease	4 (24%)	2 (12%)	Chi Square test n.s
Hyperlipidemia	16 (94%)	10 (59%)	Chi Square test *p* < 0.05
Qualifying event:			
Ischemic stroke	11 (65%)	17 (100%)	Chi Square test *p* < 0.05
TIA	6 (35%)	0 (0%)	Chi Square test *p* < 0.05
Ipsilateral ICA stenosis ≥ 50%	17 (100%)	0 (0%)	Chi Square test *p* < 0.05
Contralateral ICA stenosis ≥ 50%	6 (35%)	0 (0%)	Chi Square test *p* < 0.05
No contralateral ICA stenosis	11 (65%)	17 (100%)	Chi Square test *p* < 0.05
Ipsilateral orthograde flow direction of the supratrochlear artery	14 (82%)	17 (100%)	Chi Square test n.s.
Contralateral orthograde flow direction of the supratrochlear artery	14 (82%)	17 (100%)	Chi Square test n.s.
Corrected visual acuity in % (±SD)	Symptomatic ICA side: 89.0 (±16.4)	Ipsilateral side: 91.7 (±10.6)	Mann Whitney *U*-test n.s.
Corrected visual acuity in % (±SD)	Asymptomatic ICA side: 84.3 (±10.5)	Contralateral side: 87.8 (±8.6)	Mann Whitney *U*-test n.s.
Intraocular pressure in mmHg (±SD)	Symptomatic ICA side: 15.1 (±3.6)	Ipsilateral side: 15.4 (±3.4)	Mann Whitney *U*-test n.s.
Intraocular pressure in mmHg (±SD)	Asymptomatic ICA side: 14.3 (±3.9)	Contralateral side: 15.5 (±3.4)	Mann Whitney *U*-test n.s.
Baseline OCT and mfVEP were obtained mean ± SD days after the ischemic stroke or TIA event	6.12 ± 5.16	5.13 ± 2.47	Mann Whitney *U*-test n.s.

*Absolute numbers and percentages or median with interquartile range (IQR) are provided for demographics and risk factors, means, and standard deviations for ocular measurements. Chi Square test and interval scaled baseline parameters were compared by Mann Whitney U-test were used to compare the sCAD and the control group with microangiopathic ischemic stroke nominally scaled baseline parameters, respectively. P-values < 0.05 were considered as statistically significant, n.s. indicates no significant difference. The visual acuity and intraocular pressure did not differ significantly between the symptomatic and asymptomatic side of the sCAD group (Wilcoxon matched pairs test p = 0.117 and p = 0.138, respectively), but showed a trend toward higher visual acuity and higher intraocular pressure within normal range on the symptomatic ICA side, which were not considered as relevant*.

### Ultrasound

Only ICA stenoses ≥50% were considered as relevant and any stenosis <50% was classified as no stenosis. All 17 patients had symptomatic ipsilateral ≥50% ICA stenoses, 14 with an ipsilateral orthograde, and 3 with a retrograde flow direction of the supratrochlear artery. Out of these 17 patients, 6 also had an asymptomatic ICA stenosis ≥50% on the contralateral side. Fourteen with a contralateral orthograde and 3 with a retrograde flow direction of the supratrochlear artery. All control group patients (*N* = 17) with microangiopathic ischemic stroke had bilateral ICAs with <50% stenosis and consequently bilateral orthograde flow directions of the supratrochlear arteries. See Table 1.

### Secondary Prevention

Eight (47%) of our patients received a thrombendarterectomy of the symptomatic ICA, 7 (41%) patients carotid stenting, and 2 (12%) patients were treated with best medical treatment after the baseline measurement.

### OCT at Baseline

Complete OCT scans of both eyes were obtained from all patients and revealed no structural abnormalities in any of the retinal layers or in the pigment epithelium in any of the subjects meeting the inclusion criteria (data provided in Table S1) and of the 17 available control group patients with microangiopathic ischemic stroke.

**Figure 2:**
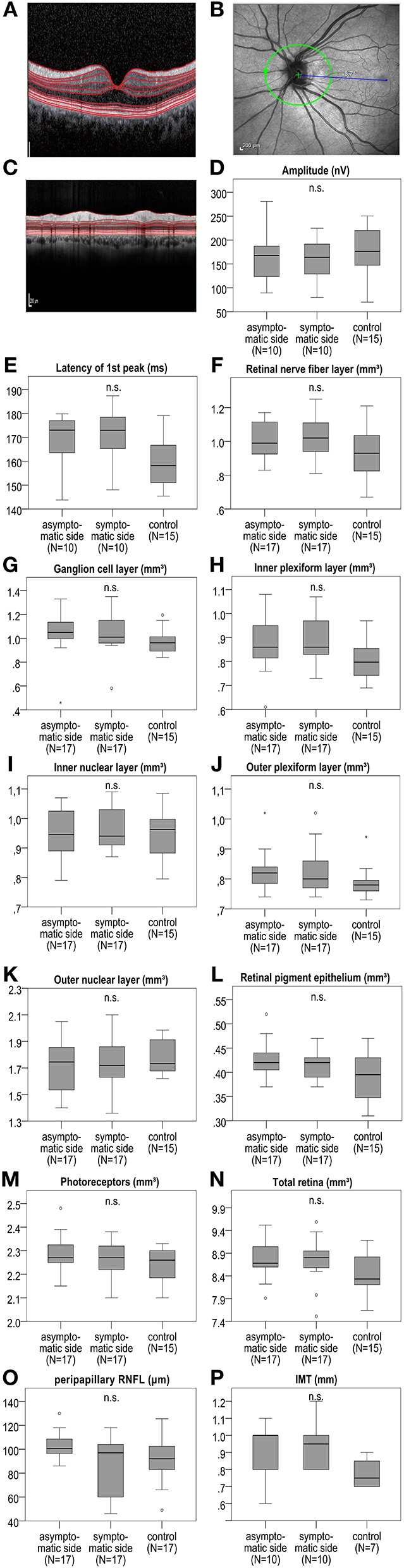
IMT, mfVEP amplitude, and first peak latency as well as macular retinal layer volume and peripapillary RNFL at baseline between the symptomatic ICA side, asymptomatic contralateral side, and the control group with microangiopathic ischemic stroke. **(A)** Shows an exemplary macular OCT layer scan. **(B)** Demonstrates an exemplary OCT scan of the peripapillary retina with the optic disc indicating the location of the peripapillary OCT ring scan by a green circle. **(C)** Shows an exemplary peripapillary OCT scan. In mfVEPs, amplitude **(D)** and first peak latency **(E)** between the symptomatic and asymptomatic ICA side, and the mean of the right and left eye of the control group were analyzed. OCT layers were measured in volume scans centered on the fovea and pRNFL was measured in peripapillary ring scans centered on the optic disc. **(F–O)** The analysis revealed no significant differences of the retinal layer volume or thickness at baseline between the symptomatic ICA side, the asymptomatic contralateral side and the mean of the right and left eye of the control group. **(P)** IMT was measured at the far wall side of both common carotid artery (CCA) 2 cm below the distal end of CCA on both sides, showing no significant difference between the symptomatic and asymptomatic ICA side, and the mean of the right and left side of the control group. The boxplots **(D–P)** demonstrate the medians of the symptomatic ICA, asymptomatic side, and of the means of the right and left side of the control group. The median is indicated by the dark line in the middle of the box, the IQR by the box, and the minimum and maximum values by whiskers (excluding outliers). Outliers defined as values 1.5 to 3.0 times outside the IQR are presented as circles and extreme outliers defined as values of more than 3.0 times outside the IQR are presented as asterisks. N.s. indicates no significant difference. *P*-values < 0.05 were considered as statistically significant (Wilcoxon matched pairs test comparing the symptomatic and asymptomatic side of the sCAD patients and Mann Whitney *U*-test comparing the symptomatic and asymptomatic side to the mean of the right and left eyes of the control group; all analyses were performed with Bonferroni correction for multiple testing).

At baseline, we observed no significant difference of the measured retinal layers (Figure 2 and Table S1) in the total cohort of 17 patients between the symptomatic ICA stenosis side, the contralateral side, and the mean of the right and left eyes of the 15 available control group patients with microangiopathic ischemic stroke for the macular OCT scans and 17 control group patients with microangiopathic ischemic stroke for the peripapillary retinal nerve fiber layer (pRNFL). In addition, a subgroup analysis at baseline revealed no significant difference of the retinal layers between the ipsilateral symptomatic side of 11 patients with ICA stenoses ≥50% and the contralateral side with no ICA stenosis. Likewise, for the 6 patients with bilateral ICA stenoses ≥50%, no significant differences were detected between the ipsilateral symptomatic and the contralateral asymptomatic side (Tables S2, S3). Furthermore, the orthograde or retrograde flow direction of the supratrochlear artery had no significant influence on the measured retinal layers (Tables S4–S6).

### Longitudinal Changes in OCT

**Figure 3:**
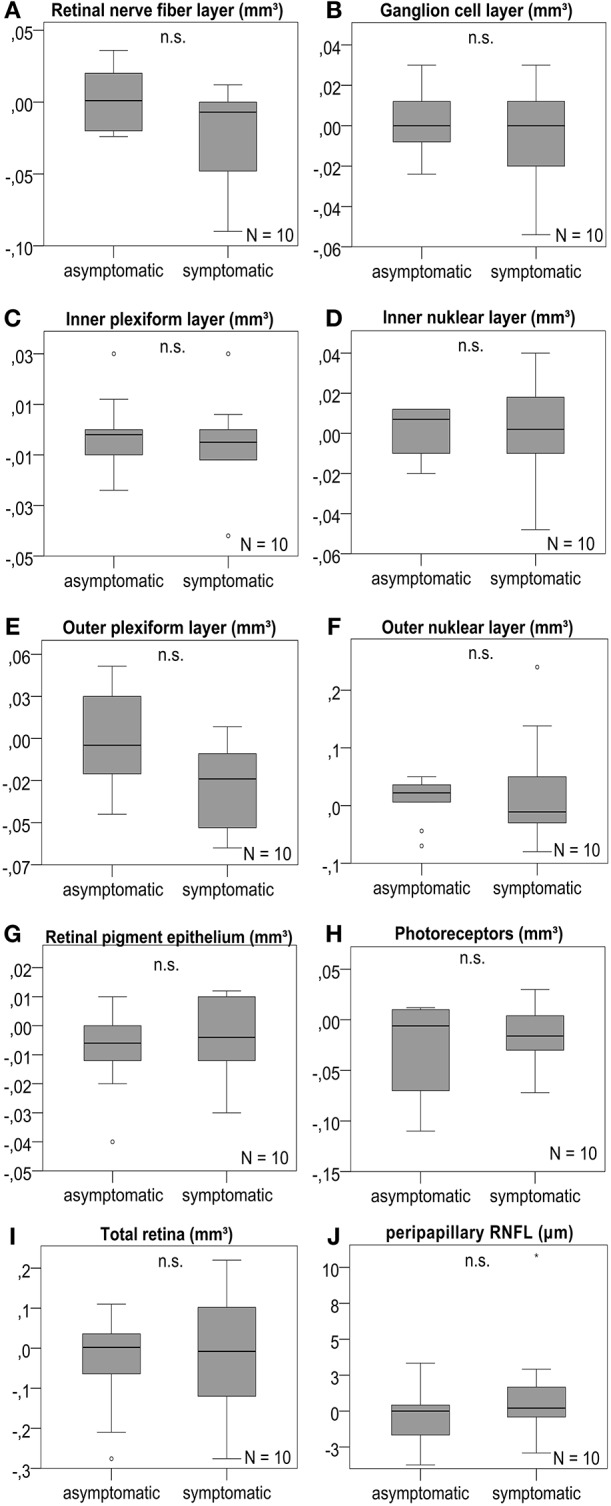
Retinal layer differences after 4 months. OCT layers were measured in volume scans centered on the fovea and pRNFL was measured in peripapillary ring scans of the optic disc. **(A–J)** The analysis revealed no significant change of the retinal layer volume or pRNFL thickness after 4 months between the symptomatic ICA side and the asymptomatic contralateral side; boxplots are demonstrated for the different retinal layers with n.s. indicating no significant difference. *P*-values < 0.05 were considered as statistically significant (Wilcoxon matched pairs test with Bonferroni correction for multiple testing). The horizontal lines in the middle of the boxplots demonstrate the medians of the symptomatic and asymtpomatic ICA side. The IQR is presented by the box, and the minimum and maximum values by whiskers (excluding outliers). Outliers defined as values 1.5 to 3.0 times outside the IQR are presented as circles and extreme outliers defined as values of more than 3.0 times outside the IQR are presented as asterisks.

We studied longitudinal retinal changes over 4 months compared to baseline. For 10 patients available for analysis, we found no significant change of volumes or thickness of any retinal layer (Figure 3 and Table S7). The subgroup analysis considering the 7 patients without contralateral ICA stenosis revealed no significant difference, as well (Table S8).

### MfVEP at Baseline

We obtained mfVEP from 10 patients at baseline. No significant changes in amplitude or first peak latency were detected between the ipsilateral and contralateral eye of symptomatic ICA stenosis patients as well as the mean of the right and left eyes of the control group with 15 available microangiopathic ischemic stroke patients (Figure 2 and Table S9).

Furthermore, no significant differences were detected between the ipsilateral eye and the contralateral eye of the subgroup of 6 patients without contralateral ICA stenosis or of the subgroup of 4 patients with contralateral ICA stenoses ≥50%, as well as for 15 eyes with orthograde flow direction of the supratrochlear artery compared to 5 eyes with retrograde flow direction of the supratrochlear artery (Table S9).

### IMT at Baseline

In the 10 sCAD patients available for analysis, no significant difference of IMT thickness between the ipsi- and contralateral CCA was detected (ipsilateral mean IMT: 0.95 ± 0.11785 mm SD; contralateral mean IMT contralateral: 0.89 ± 0.15951 mm SD, *p*-value: n.s. with Wilcoxon matched pairs test and Bonferroni correction for multiple testing). Similarly no differences were observed when comparing to the mean of the right and left IMT (0.76 ± 0.07319 mm) of the 7 control group CCAs (Mann Whitney *U*-test with Bonferroni correction for multiple testing). Furthermore, no correlation (Spearman correlation with Bonferroni correction for multiple testing) between IMT and retinal layer volume or thickness in OCT or mfVEP parameters (first latency peak and amplitude) of the sCAD group was identified (data not shown).

## Discussion

We investigated if symptomatic internal carotid artery disease is associated with changes in retinal morphology detectable by OCT using a cross-sectional and a prospective study design. A strength of our study was that not only structure (OCT) but also function (mfVEP) was investigated and the longitudinal investigations, which allowed us to analyze the effect of symptomatic internal carotid artery disease at baseline and during follow up. Further advantages are the high repeatability of the methods used (OCT and mfVEP) (18, 19) and the comparison of the parameters to a control group with microangiopathic ischemic stroke and similar age, gender as well as vascular risk factor profile. Only hyperlipidemia was less frequent in the control group, which is not surprising as hyperlipidemia is a common and well-known risk factor for typical macroangiopathic cardiovascular disorders including large vessel ischemic stroke like sCAD, myocardial infarction and peripheral arterial disease (20), whereas the role of hyperlipidemia in microangiopathic ischemic stroke is more controversial (21). At baseline there was no difference in the OCT and mfVEP parameters between the symptomatic ICA, the contralateral side, and the control group with microangiopathic ischemic stroke. After 4 months no significant change of the retinal layers in OCT of the sCAD group was detected as well. In summary, we can conclude that there were no relevant differences in retinal layer thickness between eyes supplied by symptomatic stenotic internal carotid arteries and the contralateral eyes, both at baseline and after 4 months, regardless if a contralateral ICA stenosis ≥50% was present or not. Furthermore, no signs of functional impairment between the eyes were detected in mfVEPs at baseline while not enough longitudinal mfVEP data were available at follow up to yield conclusive results.

So far, there is inconsistent data on asymptomatic internal carotid artery disease. On the one hand, Heßler et al. (22) did not identify any changes in optic nerve diameter, retinal morphology, or visual functionality. On the other hand, Wang et al. (23) including a much larger sample size of 3.376 participants demonstrated a correlation of prevalence and degree of internal carotid artery stenosis with thinner RNFL and vice versa. In that study two experienced examiners scanned all study patients with rigorous quality control (23). Furthermore, in another study, focal retinal nerve fiber layer defects showed an association with previous or acute cerebrovascular ischemic stroke and vice versa (24).

The negative findings of our OCT and mfVEP analyses might partly be explained by the small sample size and different ethnicity of the group (Caucasian vs. Asian) compared to the studies of Wang et al. (23, 24). Furthermore, we investigated symptomatic CAD and not asymptomatic CAD. In order to detect small changes, bigger cohort sizes and a longer follow up periods may be required. However, we can already conclude from this small scale study that differences that would be clinically meaningful or useful in a clinical setting cannot be observed. We decided to choose intrasubject comparisons (symptomatic ICA stenosis side vs. contralateral side) and additionally intersubject comparisons (symptomatic ICA stenosis side or asymptomatic side vs. eyes from control subjects with microangiopathic ischemic stroke and similar vascular risk factor profile) for better comparability of baseline parameters and control for comorbidities like diabetes mellitus, arterial hypertension, etc.

The lack of a healthy control cohort might be regarded as a limitation of our study, but we provided a comparison with a microangiopathic ischemic stroke control group, who had similar age, gender, and vascular risk factors to more specifically address the influence of the symptomatic ICA stenosis, which revealed no significant difference of the OCT and mfVEP parameter to the sCAD group at baseline. In this context we have to acknowledge that some patients of the sCAD group had CAD ≥50% on the contralateral side. However, these eyes did not differ from contralateral eyes, which were supplied by a non-stenotic ICA, and both groups did not differ from the symptomatic side supporting our main finding that ICA stenoses have no significant impact on retinal morphology and visual system function. Further limitations of the study are the lack of retinal blood flow assessments like fluorescein angiography with the possibility to determine the time to peak of fluorescein application to the retinal fluorescein signal, OCT- angiography or laser speckle flowgraphy and the lack of functional retinal analysis by multifocal electroretinograms.

We believe that our study provides sufficient evidence that retinal layer thickness is not useful for monitoring of symptomatic ICA stenosis. To exclude subtle changes in the long term follow up further larger studies with longitudinal design including a longer follow up period as well as intrasubject and intersubject comparisons with a healthy age and gender matched control group would be needed. However, the magnitude of such subtle changes is likely to be below the accuracy of the measurements and therefore unlikely to be meaningful on a single patient level in a clinical setting.

## Data Availability

The datasets generated for this study are available on request to the corresponding author.

## Ethics Statement

This prospective observational study (Study-number 4436R) was carried out in accordance with the recommendations of the local ethics committee of Heinrich Heine University Duesseldorf with written informed consent from all subjects. All subjects gave written informed consent in accordance with the Declaration of Helsinki. The protocol was approved by the local ethics committee of Heinrich Heine University Duesseldorf.

## Author Contributions

J-IL and PA contributed to the study design, data acquisition, data analysis, drafting of the manuscript, revision of the manuscript for important intellectual content. LG contributed to the data acquisition, data analysis, drafting of the manuscript, revision of the manuscript for important intellectual content. LB and RG contributed to the data acquisition, revision of the manuscript for important intellectual content. OA, MG, SJ, and H-PH contributed to the revision of the manuscript for important intellectual content.

### Conflict of Interest Statement

Financial disclosures unrelated to the work are presented: J-IL has received honoraria for speaking/consultation from Bayer Healthcare, Boehringer Ingelheim and Daiichi-Sankyo as well as travel grants from Bayer Healthcare, Merz Pharmaceuticals, Ipsen and Allergan outside the submitted work. OA has received honoraria for speaking/consultation and travel grants from Bayer Healthcare, Biogen Idec, Chugai, Novartis, Medimmune, Merck Serono, and Teva and research grants from Bayer Healthcare, Biogen Idec, Novartis, and Teva, outside the submitted work. MG has received honoraria for speaking/consultation from Bayer Healthcare, Boehringer Ingelheim and a research grant from B. Braun, outside the submitted work. SJ has received honoraria for speaking/consultation from Bayer Healthcare, Boehringer Ingelheim, Bristol-Myers Squibb, Pfizer, Biogen, and Daiichi-Sankyo as well as travel grants from Bayer Healthcare and Daiichi-Sankyo, outside the submitted work. H-PH has outside the work presented here received fees for serving on steering or data monitoring commitees from Bayer Healthcare, Biogen, Celgene Receptos, GeNeuro, Sanofi Genzyme, Merck, Novartis, Octapharma, Teva Pharmaceuticals, MedImmune, and Roche, fees for serving on advisory boards from Biogen Idec, Sanofi Genzyme, Merck, Novartis Pharmaceuticals, Octapharma, Teva Pharmaceuticals, and Roche, and lecture fees from Biogen, Sanofi Genzyme, Merck, Novartis Pharmaceuticals, Octapharma, Teva Pharmaceuticals, MedImmune, and Roche. PA reports grants, personal fees and non-financial support from Allergan, Biogen, Ipsen, Merz Pharmaceuticals, Novartis, and Roche, personal fees and non-financial support from Bayer Healthcare, Merck, and Sanofi-Aventis/Genzyme, outside the submitted work. The remaining authors declare that the research was conducted in the absence of any commercial or financial relationships that could be construed as a potential conflict of interest.
