# Supplementary material for: No Alteration of Optical Coherence Tomography and Multifocal Visual Evoked Potentials in Eyes With Symptomatic Carotid Artery Disease
Source: Front Neurol. 2019 Jul 10;10:741. doi: 10.3389/fneur.2019.00741 (PMC6636551; doi:10.3389/fneur.2019.00741)
Supplement: Supplementary file 1 [file Data_Sheet_1.pdf]

Supplementary information

Table S1: OCT findings at baseline, the analysis considering all sCAD patients (n=17) and the control group of patients with microangiopathic ischemic stroke (n=17): Means, standard deviations and p-values are provided for each macular retinal layer volume and the peripapillary RNFL (pRNFL) thickness at baseline, comparing the side of the symptomatic ICA, the asymptomatic contralateral side and the mean of the right and left eyes of the 15 control group patients available for the macular OCT scans and the 17 control group patients available for the pRNFL, n.s. indicating no significant difference. P-values <0.05 were considered as statistically significant (Wilcoxon matched pairs test with Bonferroni correction for multiple testing comparing the symptomatic to the asymptomatic side and Mann Whitney U test with Bonferroni correction for multiple testing comparing the symptomatic and asymptomatic side to the mean of the right and left eyes of the control group).

	**Mean value symptomatic side**	**+/-SD**	**Mean value asymptomatic side**	**+/-SD**	**Mean value microangiopathic control group**	**+/-SD**	**p-value**
**RNFL (mm^3)**	1.0318	0.12822	1.0041	0.11164	0.9253	0.15037	n.s.
**GCIP (mm^3)**	1.0424	0.16851	1.0329	0.17954	0.9670	0.10380	n.s.
**IPL (mm^3)**	0.8906	0.09297	0.8788	0.10925	0.7983	0.07362	n.s.
**INL (mm^3)**	0.9618	0.07029	0.9447	0.08308	0.9520	0.07722	n.s.
**OPL (mm^3)**	0.8271	0.07752	0.8312	0.06990	0.7760	0.02779	n.s.
**ONL (mm^3)**	1.7412	0.18990	1.7147	0.18971	1.7907	0.12831	n.s.
**RPE (mm^3)**	0.4094	0.04145	0.4194	0.04616	0.3997	0.04240	n.s.
**PR (mm^3)**	2.2671	0.07864	2.2812	0.08373	2.2467	0.07235	n.s.
**TRT (mm^3)**	8.7500	0.49299	8.7188	0.38498	8.4623	0.44807	n.s.
**pRNFL (µm)**	101.0588	12.74005	102.9412	11.14378	92.2353	14.40933	n.s.

Table S2: Subgroup OCT findings at baseline, the analysis considering only patients without contralateral ICA stenosis (n=11): Mean value, standard deviation and p-value result of each macular retinal layer volume and pRNFL at baseline between the side of the symptomatic ICA and the asymptomatic contralateral without stenosis with n.s. indicating no significant difference. P-values <0.05 were considered as statistically significant (Wilcoxon matched pairs test with Bonferroni correction for multiple testing).

	**Mean value symptomatic side**	**+/-SD**	**Mean value asymptomatic side**	**+/-SD**	**p-value**

RNFL (mm³)	1.0327	0.14297	0.9964	0.11604	n.s.
GCIP (mm³)	1.0564	0.09233	1.0536	0.07553	n.s.
IPL (mm³)	0.8782	0.07167	0.8727	0.07072	n.s.
INL (mm³)	0.9591	0.07092	0.9545	0.07313	n.s.
OPL (mm³)	0.8218	0.08010	0.8336	0.07433	n.s.
ONL (mm³)	1.7355	0.14916	1.6809	0.18907	n.s
RPE (mm³)	0.4136	0.02767	0.4209	0.03961	n.s.
PR (mm³)	2.2655	0.07244	2.2800	0.08660	n.s.
TRT (mm³)	8.7318	0.34710	8.6945	0.35895	n.s.
pRNFL (µm)	103.0000	9.53939	102.7273	12.86150	n.s.

Table S3: Subgroup OCT findings at baseline, the analysis considering only patients with bilateral ICA stenoses ≥ 50% (n = 6): Means, standard deviations and p-values for each macular retinal layer volume and pRNFL at baseline are reported for patients with bilateral ≥ 50% ICA stenoses comparing the symptomatic ipsilateral and the asymptomatic contralateral side, n.s. indicating no significant difference. P-values <0.05 were considered as statistically significant (Wilcoxon matched pairs test with Bonferroni correction for multiple testing).

	**Mean value symptomatic side**	**+/-SD**	**Mean value asymptomatic side**	**+/-SD**	**p-value**
**RNFL (mm³)**	1.0300	0.10826	1.0183	0.11215	n.s.
**GCIP (mm³)**	1.0167	0.26942	0.9950	0.29845	n.s.
**IPL (mm³)**	0.9133	0.12817	0.8900	0.16721	n.s.
**INL (mm³)**	0.9667	0.07554	0.9267	0.10386	n.s.
**OPL (mm³)**	0.8367	0.07891	0.8267	0.06743	n.s.
**ONL (mm³)**	1.7517	0.26589	1.7767	0.19117	n.s.
**RPE (mm³)**	0.4017	0.06210	0.4167	0.06055	n.s.
**PR (mm³)**	2.2700	0.09633	2.2833	0.08618	n.s.
**TRT (mm³)**	8.7833	0.73124	8.7633	0.46142	n.s.

**pRNFL (µm)**	97.5000	17.71722	103.3333	8.14043	n.s.

Table S4: Subgroup OCT findings at baseline, the analysis comparing eyes with orthograde supratrochlear artery (N=28) and eyes with retrograde supratrochlear artery (n=6): Means, standard deviations and p-values for each macular retinal layer volume and pRNFL at baseline are reported, n.s. indicating no significant difference. P-values <0.05 were considered as statistically significant (Wilcoxon matched pairs test with Bonferroni correction for multiple testing).

	**Mean value of eyes with orthograde supratrochlear artery N=28**	**+/-SD**	**Mean value of eyes with retrograde supratrochlear artery N=6**	**+/-SD**	**p-value**
**RNFL (mm^3)**	1.027143	0.12033	0.9750	0.11362	n.s.
**GCIP (mm^3)**	1.0636	0.16364	0.9167	0.16801	n.s.
**IPL (mm^3)**	0.8886	0.10255	0.8667	0.09395	n.s.
**INL (mm^3)**	0.9604	0.07876	0.9200	0.05727	n.s.
**OPL (mm^3)**	0.8221	0.06866	0.8617	0.08886	n.s.
**ONL (mm^3)**	1.7225	0.17094	1.7533	0.27023	n.s.
**RPE (mm^3)**	0.4229	0.03505	0.3750	0.05992	n.s.
**PR (mm^3)**	2.2818	0.07655	2.2383	0.09517	n.s.
**TRT (mm^3)**	8.7786	0.40939	8.5283	0.53548	n.s.
**pRNFL (µm)**	103.1875	10.24963	91.3000	10.5522	n.s.

Table S5: Subgroup OCT findings at baseline, the analysis comparing eyes with retrograde supratrochlear artery on the symptomatic side (N=3) and eyes with orthograde supratrochlear artery on the asymptomatic side (n=14): Means, standard deviations and p-values for each macular retinal layer volume and pRNFL at baseline are reported, n.s. indicating no significant difference. P-values <0.05 were considered as statistically significant (Wilcoxon matched pairs test with Bonferroni correction for multiple testing).

	**Mean value symptomatic side with retrograde supratrochlear artery N=3**	**+/-SD**	**Mean value asymptomatic side with orthograde supratrochlear artery N=14**	**+/-SD**	**p-value**
**RNFL (mm^3)**	0.9233	0.08083	1.0186	0.11428	n.s.

**GCIP (mm³)**	0.8167	0.31086	1.0486	0.19536	n.s
**IPL (mm³)**	0.8100	0.18330	0.8829	0.11485	n.s.
**INL (mm³)**	0.8900	0.11136	0.9579	0.08604	n.s.
**OPL (mm³)**	0.8533	0.07767	0.8207	0.07011	n.s.
**ONL (mm³)**	1.8900	0.23516	1.7107	0.20484	n.s.
**RPE (mm³)**	0.3833	0.07506	.4264	0.04050	n.s.
**PR (mm³)**	2.2500	0.10149	2.2914	0.08273	n.s.
**TRT (mm³)**	8.5233	0.31565	8.7686	0.40354	n.s.
**pRNFL (µm)**	83.3333	16.50253	104.5000	11.27796	n.s.

Table S6: Subgroup OCT findings at baseline, the analysis comparing eyes with orthograde supratrochlear artery on the symptomatic side (N=14) and eyes with retrograde supratrochlear artery on the asymptomatic side (n=3): Means, standard deviations and p-values for each macular retinal layer volume and pRNFL at baseline are reported, n.s. indicating no significant difference. P-values <0.05 were considered as statistically significant (Wilcoxon matched pairs test with Bonferroni correction for multiple testing).

	**Mean value symptomatic side with orthograde supratrochlear artery N=14**	**+/-SD**	**Mean value asymptomatic side with retrograde supratrochlear artery N=3**	**+/-SD**	**p-value**
**RNFL (mm³)**	1.0113	0.11716	0.947500	0.085000	n.s.
**GCIP (mm³)**	1.0680	0.120783	0.980000	0.040000	n.s.
**IPL (mm³)**	0.886667	0.086987	0.875000	0.069522	n.s.
**INL (mm³)**	0.963333	0.070980	0.922500	0.073655	n.s.
**OPL (mm³)**	0.816000	0.065553	0.870000	0.074386	n.s.
**ONL (mm³)**	1.741333	0.152028	1.882500	0.206942	n.s.
**RPE (mm³)**	0.417333	0.031045	0.382500	0.049917	n.s.
**PR (mm³)**	2.272667	0.065843	2.257500	0.083417	n.s.
**TRT (mm³)**	8.778000	0.402318	8.735000	0.251064	n.s.

pRNFL	104.857143	8.356086	86,3333	19,13984	n.s.

Table S7: OCT findings at follow up with analysis considering all available patients (n=10) after 4 months. Mean values and standard deviations of the absolute changes from baseline to the 4-month follow up are demonstrated along with p-values for the comparison of the retinal parameters between the side of the symptomatic ICA and the asymptomatic contralateral side, n.s. indicating no significant difference. P-values <0.05 were considered as statistically significant (Wilcoxon matched pairs test with Bonferroni correction for multiple testing).

	**Mean change symptomatic side**	**+/-SD**	**Mean change asymptomatic side**	**+/-SD**	**p-value**
**RNFL (mm^3)**	-0.0190	0.02795	0.0019	0.01781	n.s.
**GCIP (mm^3)**	-0.0038	0.02085	0.0013	0.01370	n.s.
**IPL (mm^3)**	-0.0040	0.01434	-0.0013	0.01166	n.s.
**INL (mm^3)**	0.0010	0.02289	0.0014	0.00940	n.s.
**OPL (mm^3)**	-0.0182	0.02147	0.0038	0.02332	n.s.
**ONL (mm^3)**	0.0194	0.07659	0.0082	0.03026	n.s.
**RPE (mm^3)**	-0.0037	0.01131	-0.0064	0.01129	n.s.
**PR (mm^3)**	-0.0133	0.02649	-0.0210	0.03590	n.s.
**TRT (mm^3)**	-0.0154	0.12121	-0.0272	0.09578	n.s.
**pRNFL (µm)**	1.0000	2.98142	-0.3333	1.54760	n.s.

Table S8: Subgroup OCT findings after 4 months, the analysis considering only available patients without contralateral ICA stenosis (n=7) after 4 months. Means of the absolute change from baseline to 4 months and standard deviations are provided. p-values for comparison of the side of the symptomatic ICA artery and the asymptomatic contralateral without stenosis are indicated, n.s. indicating no significant difference. P-values <0.05 were considered as statistically significant (Wilcoxon matched pairs test with Bonferroni correction for multiple testing).

	**Mean change symptomatic side**	**+/-SD**	**Mean change asymptomatic side**	**+/-SD**	**p-value**
**RNFL (mm^3)**	-0.026971	0.030025	-0.001829	0.017827	n.s.
**GCIP (mm^3)**	-0.008229	0.023811	0.003886	0.013635	n.s.

**IPL (mm^3)**	-0.005257	0.017126	0.000914	0.012004	n.s.
**INL (mm^3)**	-0.0053	0.024101	-0.000914	0.010445	n.s.
**OPL (mm^3)**	-0.019429	0.023541	0.011429	0.024167	n.s.
**ONL (mm^3)**	0.016686	0.083225	0.002743	0.034573	n.s.
**RPE (mm^3)**	-0.004343	0.013249	-0,008229	0.013078	n.s.
**PR (mm^3)**	-0.014629	0.031359	-0,029943	0.039858	n.s.
**TRT (mm^3)**	-0.031771	0.141110	-0.045486	0.111085	n.s.
**pRNFL (µm)**	-0.142862	1.245105	0.095245	1.652320	n.s.

Table S9: MfVEP findings at baseline. Means and standard deviations as well as p-values for the comparison of mfVEP first peak latencies (ms) and amplitudes (nV) at baseline are provided comparing the side of the symptomatic ICA,the asymptomatic contralateral side and the mean of the right and left eyes of the control group with microangiopathic ischemic stroke. Analyses were performed for all available sCAD patients (n=10), for the available mean of the right and left eyes of the control group (n=15), for sCAD patients without contralateral ICA stenosis (n=6), for sCAD patients with bilateral ICA stenosis ≥ 50% (n=4) and for eyes of sCAD patients with orthograde (N=15) compared to retrograde (N=5) supratrochlear artery. N.s. indicates no significant difference and p-values <0.05 were considered as statistically significant (Wilcoxon matched pairs test with Bonferroni correction for multiple testing comparing the symptomatic to the asymptomatic side within the sCAD group and Mann Whitney U test with Bonferroni correction for multiple testing comparing the symptomatic and asymptomatic side to the mean of the right and left eyes of the control group).

	**Mean value symptomatic side**	**+/-SD**	**Mean value asymptomatic side**	**+/-SD**	**Mean value microangiopathic control group**	**+/-SD**	**p-value**
	All available patients, **n=10**				**n=15**		
**Amp (nV)**	157.1500	46.245	166.5010	58.419	178.1773	55.174	n.s
**1st peak latency (ms)**	171.1030	11.163	169.4000	11.188	158.9197	10.514	n.s
**n=6**	Analysis considering only patients without contralateral ICA stenosis				**p-value**		
**Amp (nV)**	164.2483	49.69390	182.5533	66.77128	n.s		
**1st peak**	170.7783	13.30713	168.3850	13.25131	n.s		

**latency (ms)**					
**N=4**	Analysis considering only patients with bilateral ICA stenosis ≥ 50%				**p-value**
**Amp (nV)**	146.5025	45.25649	142.4225	38.97716	n.s
**1st peak latency (ms)**	171.5900	8.84158	170.9225	8.81623	n.s
	**Mean value orthograde supratrochlear artery**	**+/-SD**	**Mean value retrograde supratrochlear**	**+/-SD**	**p-value**
**N=20**	Analysis considering only eyes (N=15) of patients with orthograde supratrochlear artery compared to eyes (N=5) of patients with retrograde supratrochlear artery				
**Amp (nV)**	162.2080	54.93349	160.6780	45.12010	n.s.
**1st peak latency (ms)**	169.8367	10.46620	171.4960	13.40443	n.s.
